# CLINICAL ASSESSMENTS AND GAIT CHARACTERISTICS BY SUBTYPE CLASSIFICATION USING TEMPORAL AND KINEMATIC SYMMETRY INDICES DURING GAIT IN PATIENTS WITH CHRONIC STROKE

**DOI:** 10.2340/jrm.v57.44623

**Published:** 2025-11-11

**Authors:** Yuichiro HOSOI, Takayuki KAMIMOTO, Kohsuke OKADA, Kohshiro HARUYAMA, Tetsuya TSUJI, Michiyuki KAWAKAMI

**Affiliations:** 1 Department of Rehabilitation Medicine, Keio University School of Medicine, Tokyo; 2 Department of Rehabilitation, Saiseikai Higashi-Kanagawa Rehabilitation Hospital, Kanagawa; 3 Department of Physical Therapy, Faculty of Health Science, Juntendo University, Tokyo, Japan

**Keywords:** stroke, gait, symmetry, classification, clinical assessment

## Abstract

**Objective:**

Gait symmetry is an important assessment indicator in patients with stroke and is classified into temporal and kinematic types. This study aimed to clarify the clinical assessments and ability characteristics of subtypes by gait symmetry through clustering analysis using symmetry indices.

**Design:**

Retrospective, cross-sectional, observational study.

**Participants:**

Fifty-nine patients with chronic stroke who could walk independently without aids during measurement, including those who habitually used an assistive device.

**Methods:**

Gait was analysed with a 3-dimensional motion analysis system and force plates. Temporal symmetry was assessed using the swing-time ratio; kinematic symmetry was assessed by the normalized cross-correlation of left–right knee angle waveforms; k-means clustering used the 2 indices; and clinical measures and comfortable gait speed were compared across subtypes.

**Results:**

Four subtypes were identified. Subtypes with high kinematic but low temporal symmetry had moderate motor paralysis, moderate gait speed, and high paretic-side propulsion, whereas subtypes with high temporal but low kinematic symmetry showed moderate motor paralysis, higher muscle tone, moderate gait speed, and lower paretic-side propulsion.

**Conclusion:**

This study provides new insights into gait symmetry in stroke and may help lay the groundwork for future studies on gait classification and rehabilitation.

## LAY ABSTRACT

Many people who have had a stroke have difficulty walking, often because one leg moves differently from the other. This study examined 59 people with chronic stroke to better understand these differences. We used a walking test to measure how similar the timing and movement of the legs were during walking. Based on these measurements, we identified 4 distinct types of walking patterns. Some individuals showed movements that were quite similar on both sides, but still walked slowly, whereas others walked faster, but had greater differences between the 2 legs. These differences were also associated with other issues, such as muscle stiffness and weakness. Understanding these walking patterns can help therapists select more appropriate exercises for each person. By tailoring rehabilitation to the specific type of walking problem, we hope to improve walking ability and increase independence in daily life.

Gait impairments are common in patients with stroke and can lead to reduced gait speed, endurance, and independence (1). Moreover, the incidence of falls within 1 year following stroke has been reported to be as high as 73% (2). Therefore, improving gait ability is a critical therapeutic goal in stroke rehabilitation (1).

A defining characteristic of gait impairment in patients with stroke is the asymmetry observed in both temporal and kinematic parameters (3). Temporal gait symmetry is typically assessed using the swing phase time ratio, which represents the relative duration of the swing phase between the paretic and non-paretic limbs (4, 5). Kinematic gait symmetry, in contrast, is evaluated by comparing the similarity of joint motion waveforms, such as knee joint kinematics, throughout the gait cycle using normalized cross-correlation coefficients (4, 5). These symmetry measures are closely related to motor function and gait performance in patients with stroke (6, 7) and have frequently been used as key assessment tools in rehabilitation research (8–10).

Although temporal and kinematic symmetry indices have been studied individually, their interrelationship remains unclear. Some studies have reported a correlation between these 2 measures (7), whereas others have observed instances in which temporal symmetry was impaired despite preserved kinematic symmetry, and vice versa (11, 12). Understanding the relationship between these symmetry indices is crucial for tailoring rehabilitation strategies, as distinct asymmetry patterns may necessitate different therapeutic approaches.

Recent advancements in data-driven analyses have enabled the classification of gait patterns in patients with stroke using clustering approaches based on various gait parameters, such as joint kinematics from motion-capture data and spatiotemporal variables including gait speed and step time (13, 14). In addition, several studies have applied clustering based on symmetry indices, particularly temporal symmetry measures such as swing-time or step-time ratios, to characterize gait subtypes after stroke (15). However, these studies have generally examined either temporal symmetry or kinematic features in isolation and, to the best of our knowledge, no study has yet investigated the combined characteristics or interrelationships of temporal and kinematic symmetry indices in classifying gait patterns. A comprehensive analysis integrating both indices may provide a more detailed understanding of gait subgroups in patients with stroke.

This preliminary study aimed to classify subgroups of patients with stroke based on both temporal and kinematic gait symmetry indices using clustering analysis. In addition, the motor function and gait characteristics of each subgroup were examined. The findings of this study will contribute to a more nuanced understanding of gait symmetry in patients with stroke and may provide a foundation for developing targeted rehabilitation interventions. We hypothesized that symmetry-based classification of gait patterns is associated with differences in clinical assessment scores in patients with chronic stroke.

## METHODS

### Participants

Participants with hemiplegia following a cerebrovascular accident and admitted to Keio University Hospital between May 2019 and March 2023 were enrolled in this retrospective, cross-sectional, observational study. The inclusion criteria were as follows: (1) diagnosis of a stroke causing unilateral sensorimotor impairment located in the cerebral hemisphere; (2) absence of hemianopsia; (3) no severe cognitive or language impairment; (4) no evidence of neglect; (5) more than 6 months since the stroke; and (6) ability to walk independently for at least 10 metres without the use of any aids or assistive devices. Patients’ demographic characteristics, affected side, and time since onset were collected. Participants with missing gait data or incomplete motion-capture recordings were excluded from the analysis (*n* = 7). The most common reasons for exclusion were marker occlusion or incomplete ground reaction force data.

Although the inclusion criterion required the ability to walk independently for at least 10 m without the use of aids or assistive devices during measurement, information regarding the habitual use of walking aids was also recorded. Several participants regularly used an ankle–foot orthosis (AFO). However, all gait analyses were conducted barefoot with no assistive devices or aids as part of the standard motion analysis protocol (7) to ensure standardized kinematic assessment and minimize mechanical interference.

This study was approved by the Medical Ethics Committee of Keio University (approval number: 20241171) and conducted in accordance with the Declaration of Helsinki. Information concerning the study was disclosed on the Keio University Hospital website. Following the opt-out procedure approved by the ethics committee, written, informed consent was waived. This cross-sectional observational study was reported in accordance with the STROBE (Strengthening the Reporting of Observational Studies in Epidemiology) guidelines.

### Procedure and data collection

*Kinematic and kinetic data during overground gait*

A 3-dimensional (3D) motion capture system (Vicon, Vicon Motion Systems, Oxford, UK) was used for gait analysis. The reflective marker sets were chosen according to the plug-in gait lower-body model. Sixteen markers were attached to anatomical landmarks on both sides (8 per side): anterior superior iliac spine, posterior superior iliac spine, thigh, knee, tibia, ankle, toe, and heel. Motion data were sampled at a frequency of 100 Hz. A total of 5 gait cycles were measured. The examination was performed with the patient barefoot and without using an assistive device. Two force plates (MG1120, Anima, Tokyo, Japan) installed on the left and right sides recorded the ground reaction force (GRF) during gait at a comfortable speed without shoes over 5 gait cycles (sampling rate 100 Hz).

All analyses were performed using in-house code in MATLAB (version R2022a; The MathWorks, Natick, MA, USA). The mean of 5 stride data was normalized to the percentage of the gait cycle. Initial contact and toe-off were identified visually from the heel and toe marker trajectories, following previously reported kinematics-based methods (16, 17). To ensure reproducibility and minimize observer bias, the identified gait events were rechecked on the lower-limb animation generated using a custom MATLAB script by 2 experienced raters independently.

### Kinematic and kinetic outcome measures

Kinematic and kinetic data were analysed using MATLAB software (MathWorks). Both sets of data were time-normalized for each gait cycle. The mean value of the joint angle of the total gait cycle was calculated for each time point (0–100%, a total of 101 points). Furthermore, to evaluate temporal gait asymmetry (swing time ratio; SR) (3), the symmetry ratio was derived from the recorded swing times in milliseconds. SR is defined as affected swing time divided by non-affected swing time (3), with a value of 1 being symmetrical; the larger it gets, the more asymmetrical the gait. Further, the normalized cross-correlation (NCC) was calculated based on the waveform of the knee joint angle of the lower extremity during gait, which was used to assess kinematic gait asymmetry (4). The knee joint was selected as the representative variable for kinematic symmetry analysis because its motion is closely associated with paretic propulsion after stroke (18) and was frequently used to characterize post-stroke gait patterns, such as stiff-knee gait, in previous classification studies (19, 20). In this study, the NCC of the knee joint was used as an indicator of kinematic symmetry. The average NCC of the 5 gait cycles was then calculated. The NCC is expressed as a value from 0 to 1. A value closer to 1 indicates a stronger correlation between the nonaffected and affected sides (i.e., kinematically symmetrical gait) (4).

GRF data were raw data-smoothed using a 10-Hz Butterworth filter and normalized to bodyweight (%BW). Paretic propulsion (%Pp) was calculated as the proportion of the total forward propulsion impulse generated by the paretic limb, using the following formula:

%Pp = (Paretic side propulsion impulse)/(Paretic side + Non-paretic side propulsion impulse) × 100

A value of 50% indicates perfect symmetry. This definition follows previous studies of post-stroke gait propulsion (21).

### Clinical outcome measures

Clinical assessments included the Fugl-Meyer Assessment-Lower Extremity (FMA-LE), the Modified Ashworth Scale (MAS), the Stroke Impairment Assessment Set (SIAS) sensory score, and the Trunk Impairment Scale (TIS). Gait ability assessments included comfortable gait speed (CGS).

The FMA is widely used to evaluate motor function. The FMA-LE assesses movement, reflexes, speed, and coordination (22, 23). The maximum score is 34, with higher scores indicating better function.

The MAS comprises a 5-level scale to examine joint spasticity during passive muscle stretching (24). Only the ankle plantar flexors were assessed according to the assessment manual from a previous study. Score 1+ was transformed to 2, and scores 2, 3, and 4 were converted to 3, 4, and 5, respectively.

The SIAS has been used to assess hemiplegia in stroke and validated for internal consistency and predictive validity. This study used the SIAS sensory score to evaluate the tactile and position sensations of the dorsal foot and articulation of the great toe on a 4-point scale from 0 to 3, with higher values indicating normal function (25, 26).

The TIS was developed as an evaluation tool to measure trunk function in patients with stroke, including quality of movement and execution time (27). It consists of a 23-point scale, with higher scores indicating greater trunk capacity.

CGS is a method of assessing gait ability that has been used to evaluate a variety of conditions in patients with stroke (28). In the present study, an acceleration distance of 3 m and a deceleration distance of 3 m were set up at the front and rear ends, respectively, of the 10-m track, and gait speed was calculated by measuring gait time in the 10-m middle section, excluding the acceleration and deceleration areas, with a digital stopwatch, and the average of 2 trials was used (29).

### Clustering using symmetry indices for gait

The symmetry indices SR and NCC during walking were used to classify participants using the k-means clustering method (30, 31). First, the optimal number of clusters was determined using the elbow method, in accordance with previous studies (31, 32). In addition, the optimal number of clusters was further confirmed using the silhouette coefficient (33), which evaluates both cluster compactness and separation. K-means clustering was implemented using the k-means++ initialization method, with 10 random restarts (n_init = 10) and a maximum of 300 iterations per run until convergence, executed in Python (version 3.1.1; https://www.python.org/) with scikit-learn (version 1.3.0; https://scikit-learn.org/). To assess the internal stability of the clustering solution, internal validation analyses were performed using the Adjusted Rand Index (ARI) and Jaccard similarity (34, 35). The ARI was computed across 50 random initializations to evaluate reproducibility under different initial conditions, whereas Jaccard similarity was calculated across 50 iterations of 80% subsampling to assess robustness to data perturbation. Higher values of these indices indicate greater stability of the clustering solution.

### Data and statistical analysis

The Shapiro–Wilk test was used to assess the normality of the data, and as the data were not normally distributed, nonparametric tests were used. Spearman’s rank correlation coefficient was used to examine the association between SR, a temporal symmetry index, and NCC, a kinematic symmetry index. Differences in basic attributes, clinical outcome measures, and gait parameters among clusters were examined using the χ^2^ test and the Kruskal–Wallis test, with Dunn’s test with Bonferroni adjustment for multiple comparisons. All statistical analyses were conducted using IBM SPSS Statistics version 28.0 (IBM Corp, Armonk, NY, USA). Values of *p* < 0.05 were considered significant.

## RESULTS

A total of 59 participants with chronic stroke were enrolled in this study. Their demographic information, clinical assessments, and gait parameters are presented in Tables I and II.

**Table I:** Characteristics of all participants and the cluster groups

Factor	Overall (*n* = 59) Median (IQR)	Cluster 1 (*n* = 22) Median (IQR)	Cluster 2 (*n* = 19) Median (IQR)	Cluster 3 (*n* = 10) Median (IQR)	Cluster 4 (*n* = 8 Median (IQR))	*p*-value
Age, years	53.00 (48.00–60.00)	54.00 (39.00–63.25)	53.00 (49.00–61.00)	53.00 (48.75–57.00)	55.00 (47.25–68.75)	*p* = 0.942
Sex, male/female	40/19	14/8	12/7	7/3	7/1	*p* = 0.322
Height, cm	165.80 (149.40–171.10)	165.15 (159.08–171.30)	164.50 (156.80–169.90)	168.90 (159.67–172.02)	166.20 (163.20–171.10)	*p* = 0.688
Weight, kg	61.55 (53.90–69.85)	60.80 (54.75–68.90)	64.80 (56.30–71.50)	65.05 (53.75–78.80)	57.00 (52.35–71.53)	*p* = 0.723
Time after onset, months	37.00 (23.50–68.00)	25.50 (19.25–51.25)	38.00 (29.50–90.50)	40.00 (29.50–92.75)	35.50 (27.25–85.75)	*p* = 0.872
Paretic side, right/left	26/33	8/14	6/13	7/3	5/3	*p* = 0.644
Walking aids, no use/use	22/37	16/6	4/15	2/8	0/8	*p* = 0.149
FMA-L/E, points	25.00 (21.00–29.00)	29.00 (27.00–30.25)	23.00 (21.00–25.00)	24.50 (22.75–28.25)	18.00 (10.25–20.75)	1 vs 2: *p* < 0.001, 1 vs 3: *p* = 0.034, 1 vs 4: *p* < 0.001, 2 vs 3: *p* = 0.154, 2 vs 4: *p* = 0.100, 3 vs 4: *p* < 0.001
MAS-gastrocnemius, points	2.00 (1.00–2.00)	1.00 (0.00–2.00)	1.00 (1.00–2.00)	2.00 (2.00–3.00)	2.50 (2.00–3.00)	1 vs 2: *p* = 1.000, 1 vs 3: *p* = 0.134, 1 vs 4: *p* = 0.023, 2 vs 3: *p* = 0.019, 2 vs 4: *p* = 0.003, 3 vs 4: *p* = 1.000
SIAS-touch, points	2.00 (2.00–3.00)	2.00 (2.00–3.00)	2.00 (2.00–3.00)	2.00 (1.00–3.00)	2.50 (2.00–3.00)	*p* = 0.765
SIAS-position, points	3.00 (2.00–3.00)	3.00 (2.00–3.00)	3.00 (2.00–3.00)	3.00 (1.00–3.00)	3.00 (1.00–3.00)	*p* = 0.919
TIS, points	18.00 (16.00–20.00)	18.00 (17.00–19.25)	17.00 (13.00–19.00)	19.50 (17.50–20.25)	13.00 (11.75–17.75)	1 vs 2: *p* = 0.129, 1 vs 3: *p* = 0.434, 1 vs 4: *p* = 0.019, 2 vs 3: *p* = 0.027, 2 vs 4: *p* = 0.239, 3 vs 4: *p* = 0.009

Statistical comparisons were performed using the Kruskal–Wallis test, and pairwise post hoc comparisons were conducted using Dunn’s test with Bonferroni adjustment for multiple comparisons. Reported *p*-values are Bonferroni-adjusted.

Walking aids indicate habitual use in daily life, not device use during gait analysis.

IQR: interquartile range; FMA-L/E: Fugl-Meyer Assessment Lower Extremity Subscale; MAS: Modified Ashworth Scale; SIAS: Stroke Impairment Assessment Set; TIS: Trunk Impairment Scale.

**Table II:** Gait ability assessment of the cluster groups

Gait ability parameter	Overall (*n* = 59) Median (IQR)	Cluster 1 (*n* = 22) Median (IQR)	Cluster 2 (*n* = 19) Median (IQR)	Cluster 3 (*n* = 10) Median (IQR)	Cluster 4 (*n* = 8) Median (IQR)	*p*-value
Comfortable gait speed, m/s	0.86 (0.64–1.12)	0.92 (0.75–1.13)	0.61 (0.52–0.73)	0.65 (0.36–0.82)	0.38 (0.33–0.49)	1 vs 2: *p* < 0.001, 1 vs 3: *p* < 0.001, 1 vs 4: *p* < 0.001, 2 vs 3: *p* = 0.794, 2 vs 4: *p* = 0.022, 3 vs 4: *p* = 0.073
Swing time ratio	1.18 (1.04–1.34)	1.03 (1.02–1.06)	1.31 (1.23–1.35)	1.13 (1.07–1.18)	1.75 (1.70–1.97)	1 vs 2: *p* < 0.001, 1 vs 3: *p* = 0.133, 1 vs 4: *p* < 0.001, 2 vs 3: *p* < 0.001, 2 vs 4: *p* = 0.064, 3 vs 4: *p* < 0.001
Normalized cross-correlation – knee joint	0.88 (0.75–0.95)	0.96 (0.93–0.97)	0.88 (0.84–0.94)	0.73 (0.56–0.75)	0.61 (0.46–0.77)	1 vs 2: *p* = 0.048, 1 vs 3: *p* < 0.001, 1 vs 4: *p* < 0.001, 2 vs 3: *p* < 0.001, 2 vs 4: *p* < 0.001, 3 vs 4: *p* = 0.984
% Paretic propulsion	31.76 (22.37–42.41)	39.70 (30.56–46.56)	33.50 (24.60–43.39)	22.33 (19.67–26.02)	23.28 (20.49–27.52)	1 vs 2: *p* = 0.124, 1 vs 3: *p* = 0.002, 1 vs 4: *p* = 0.044, 2 vs 3: *p* = 0.028, 2 vs 4: *p* = 0.063, 3 vs 4: *p* = 0.994

Statistical comparisons were performed using the Kruskal–Wallis test, and pairwise post hoc comparisons were conducted using Dunn’s test with Bonferroni adjustment for multiple comparisons.

Reported *p*-values are Bonferroni-adjusted.

IQR: interquartile range.

### Clustering using symmetry indices for gait

**Fig. 1:**
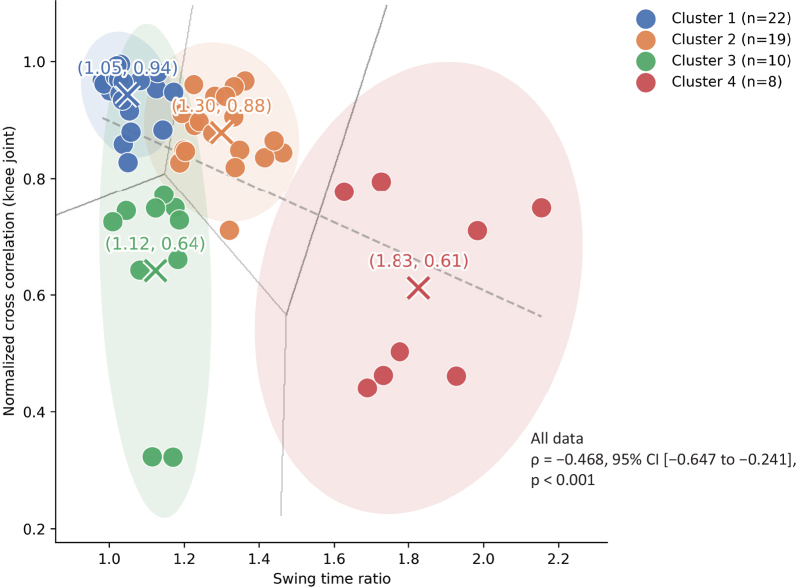
Results of cluster analysis of kinematic symmetry (normalized cross-correlation) and temporal symmetry (swing time ratio) using the k-means method. Participants were classified into 4 clusters according to the swing time ratio (SR) and the normalized cross-correlation (NCC) of the knee joint. Coloured data points represent individual participants, and shaded ellipses indicate 95% confidence regions. Cluster centroids (“×”) and their mean coordinates are shown. Grey lines represent Voronoi boundaries between clusters, and the dashed grey line indicates the regression line between SR and NCC.

A significant negative correlation was observed between SR, representing temporal symmetry, and NCC, representing kinematic symmetry (ρ = −0.468, 95% confidence interval (CI) [−0.647 to −0.241], *p* < 0.001). However, the scatter plot indicated considerable variability in the relationship between these 2 indices. Clustering analysis using the k-means method identified 4 clusters, as shown in Fig. 1. In addition, to visualize the relationships between gait speed and the symmetry indices, scatter plots showing the relationships between gait speed and SR, between gait speed and NCC, and the distribution of gait speed across clusters are provided in Figs. S1 and S2. Clusters exhibiting more symmetric gait patterns tended to have higher gait speeds; however, there was considerable overlap among clusters, suggesting that differences in symmetry-related subtypes cannot be fully explained by gait speed alone. The optimal number of clusters was supported by the elbow method and further confirmed by the silhouette coefficient (0.51), indicating moderate separation among clusters. In addition, the elbow plot used to determine the optimal number of clusters has been provided in Fig. S3 for reference. Internal validation indicated that the clustering solution was reproducible and showed acceptable robustness (ARI = 1.000; Jaccard similarity = 0.79 [95% CI: 0.56–1.00]).

Significant differences in SR were observed among clusters. Cluster 4 had significantly higher SR than Clusters 1 and 3, and Cluster 2 had significantly higher SR than Clusters 1 and 3, indicating greater asymmetry in temporal symmetry. Similarly, NCC showed significant differences among clusters. Clusters 3 and 4 had significantly lower NCC than Clusters 1 and 2, and Cluster 2 had significantly lower NCC than Cluster 1, indicating asymmetry in kinematic factors (see Table II). Based on these results, the clusters were categorized as follows: Cluster 1 had high temporal and kinematic symmetries; Cluster 2 had high kinematic symmetry, but low temporal symmetry; Cluster 3 had high temporal symmetry, but low kinematic symmetry; and Cluster 4 had low temporal and kinematic symmetries.

### Clinical and gait characteristics

Clinical assessments and gait performance differed among clusters, as shown in Tables I and II. Effect sizes for all between-cluster comparisons are summarized in Table SI to aid interpretation of the statistical results. No significant differences were found in the SIAS sensory score among clusters. However, Cluster 1 had significantly higher FMA-LE scores than the other clusters. MAS scores were lower in Cluster 1 than in Clusters 3 and 4, and TIS scores were higher in Cluster 1 than in Cluster 4. In Cluster 2, MAS was significantly lower than in Clusters 3 and 4, and the TIS score was significantly lower than in Cluster 3. Cluster 3 showed significantly higher FMA-LE scores than Cluster 4, and TIS scores were also significantly higher in Cluster 3 than in Cluster 4.

Regarding gait performance, CGS was significantly higher in Cluster 1 than in the other clusters, and %Pp was significantly higher in Cluster 1 than in Clusters 3 and 4. Cluster 2 had significantly higher CGS than Cluster 4, and %Pp was significantly higher in Cluster 2 than in Cluster 3.

Based on these findings, each cluster exhibited distinct characteristics. Cluster 1 demonstrated low muscle tone, mild motor impairment, high gait speed, and high paretic propulsion. Cluster 2 showed moderate motor impairment and low muscle tone, with moderate gait speed, but high paretic propulsion. Cluster 3 had moderate motor impairment and high muscle tone, with moderate gait speed and low paretic propulsion. Cluster 4 exhibited severe motor impairment and high muscle tone, with low gait speed and low paretic propulsion.

## DISCUSSION

This study aimed to clarify the clinical assessments and ability characteristics of the subtypes based on symmetry during gait using the interrelationship between spatiotemporal symmetry indices and kinematic symmetry indices as symmetry indices during gait, as well as clustering analysis. The clusters based on symmetry indices during gait were classified into 4 groups: Cluster 1, which had high temporal and kinematic symmetries; Cluster 2, which had high kinematic symmetry, but low temporal symmetry; Cluster 3, which had high temporal symmetry, but low kinematic symmetry; and Cluster 4, which had low temporal and kinematic symmetries. Furthermore, the clinical assessments and gait ability characteristics of each cluster showed that there were clusters with asymmetry in the time factor and clusters with asymmetry in the kinematic factor, even though the motor paralysis and gait speed were similar, and the results of the clinical assessment and gait ability differed for each cluster. The findings of this study are the first to be obtained in a study of gait symmetry in patients with stroke.

In the present study, when motor paralysis was moderate and the gait speed was moderate, Clusters 2 and 3 showed low temporal symmetry and low kinematic symmetry, respectively. Though the relationship between gait speed and motor paralysis has been clarified in a previous study (36), the present study suggested that, in patients with stroke who have moderate motor paralysis, the compensatory strategies used to maintain gait speed may differ. Although the symmetry of the time factor was low in Cluster 2, the ratio of paretic propulsion was similar to that in Cluster 1, suggesting that the asymmetry between paretic propulsion and the time factor may not be directly related. However, paretic propulsion was significantly lower in Cluster 3 than in Clusters 1 and 2, indicating that it was difficult to generate paretic propulsion and suggesting that gait speed may be achieved through a compensatory strategy using abnormal gait patterns. Previous research has reported a relationship between propulsion during gait and kinematic indicators (37), and it has also been reported that patients with stroke show little improvement in kinematic indicators, despite improvements in gait speed related to paretic propulsion (38, 39). Therefore, previous studies have reported that patients with stroke are unable to generate the propulsion necessary to increase gait speed, and they instead use compensatory strategies to maintain gait speed in abnormal gait patterns (40). Therefore, the results of the present study are considered to support the results of previous studies.

Furthermore, Cluster 2, which had low temporal symmetry, showed more severe trunk dysfunction than the other clusters. It has been reported that trunk function is related to gait speed and symmetry, and it has been shown that it contributes to stability during the single stance phase of gait (41). Therefore, it was thought that, if there is a trunk function disorder, there is a decrease in the stability of the single stance phase during gait, and as a result the symmetry of the time factor may be decreased.

However, it was found that clusters with reduced symmetry of kinematic factors showed abnormal muscle tone and reduced paretic propulsion compared with other clusters. It has been reported that abnormal gait patterns and abnormal muscle tone are related (42), and in particular it has been reported that co-contraction of muscles during gait is related to joint motion and paretic propulsion during gait (43, 44). These findings are thought to support the results obtained in the present study. However, the present study did not analyse muscle activity during gait, and some points are insufficient for discussion. Thus, there is a need to further investigate this, including muscle activity, in the future.

Treadmill gait training (45) and dynamic balance training (46) are cited as effective interventions for asymmetries in the time factor as rehabilitation for gait asymmetries in patients with stroke. Botulinum toxin treatment (47) and gait training using a gait-assist robot (48) have been reported as effective interventions for asymmetries in kinematic factors, suggesting that the approaches to each symmetry are different. Thus, the findings of the present study provide preliminary insights into gait asymmetry in patients with stroke and may serve as basic data for future research on post-stroke gait characteristics.

### Limitations

Several limitations should be noted in this study. First, although gait speed may affect both SR and NCC, it was treated as an outcome variable rather than as a covariate because the purpose of this study was to examine differences in gait performance among symmetry-related subtypes. The observed associations between gait speed and symmetry indices suggest possible interdependence among these parameters; therefore, future studies with larger cohorts should further explore this relationship, for example, through stratified analyses by gait speed. Second, differences in cluster sizes may have affected the stability of between-group comparisons; thus, the findings should be interpreted with caution. Third, gait indices were limited to kinematic parameters and did not account for other factors such as muscle activity or joint moments. Moreover, gait analyses were conducted barefoot and without shoes, orthoses, or walking aids to ensure standardized kinematic assessment, which may limit generalizability to real-world walking. A more comprehensive understanding of gait ability within each cluster and its relationship to clinical assessments can be achieved by incorporating additional variables, such as electromyography, kinetic data, and real-world walking conditions. Finally, because this study included only individuals with chronic stroke who were able to walk independently, caution should be exercised when generalizing the findings to individuals with different levels of impairment or in acute/subacute phases of stroke recovery.

### Conclusion

This study highlights the presence of distinct subtypes of gait asymmetry in patients with chronic stroke and their associations with clinical assessments. These findings may provide preliminary insights and serve as basic data for future research on post-stroke gait characteristics.

## Supplementary Material




