# Supplementary material for: CLINICAL ASSESSMENTS AND GAIT CHARACTERISTICS BY SUBTYPE CLASSIFICATION USING TEMPORAL AND KINEMATIC SYMMETRY INDICES DURING GAIT IN PATIENTS WITH CHRONIC STROKE
Source: J Rehabil Med. 2025 Nov 11;57:44623. doi: 10.2340/jrm.v57.44623 (PMC12619075; doi:10.2340/jrm.v57.44623)
Supplement: Supplementary file 2 [file JRM-57-44623-s2.pdf]

Supplementary material has been published as submitted. It has not been copyedited, or typeset by Journal of Rehabilitation Medicine

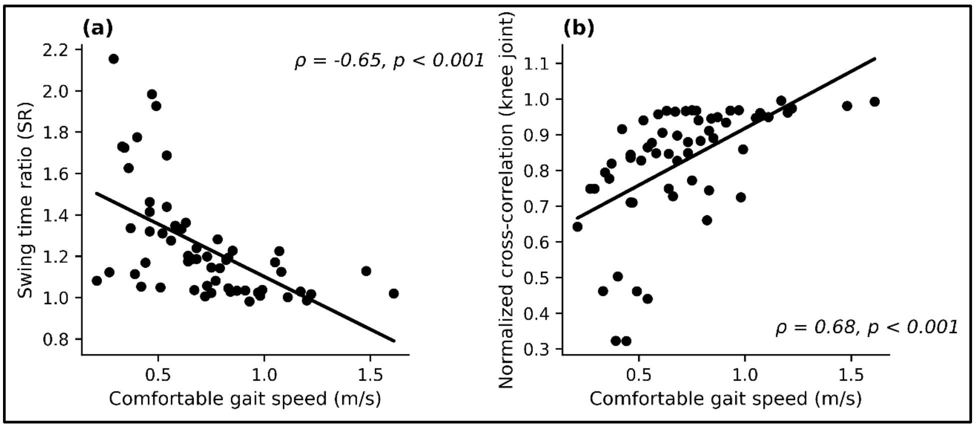

Figure S1. Scatter plots showing the relationships between comfortable gait speed (CGS) and gait symmetry indices.

(a) Comfortable gait speed (CGS) versus swing time ratio (SR), representing temporal symmetry.

(b) Comfortable gait speed (CGS) versus normalized cross-correlation (NCC) of the knee joint, representing kinematic symmetry.

Each plot includes the regression line and Spearman's correlation coefficient (ρ).

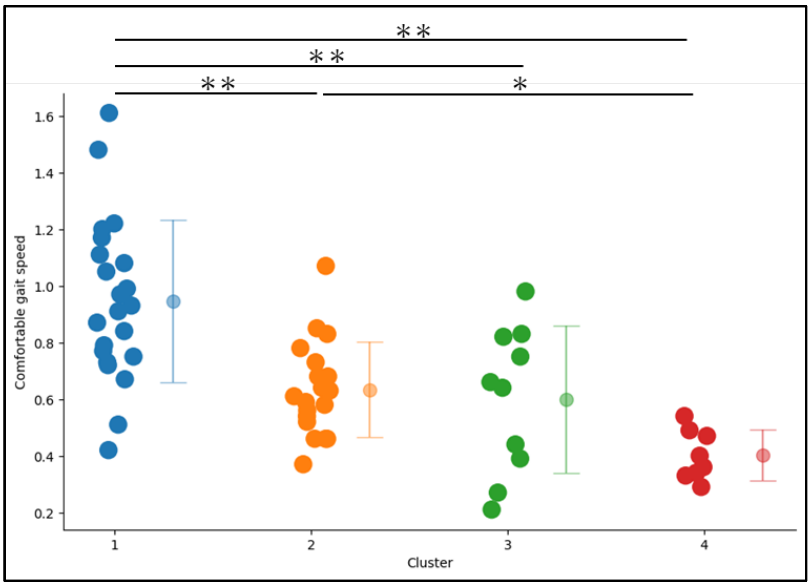

Figure S2. Scatter plots showing the relationships between comfortable gait speed (CGS) and gait symmetry indices.

Mean ± SD of comfortable gait speed (CGS) for each cluster identified by combined temporal and kinematic symmetry indices. *: $p < 0.05$, **: $p < 0.001$.

Horizontal bars indicate significant differences between clusters based on Dunn's test with Bonferroni adjustment.

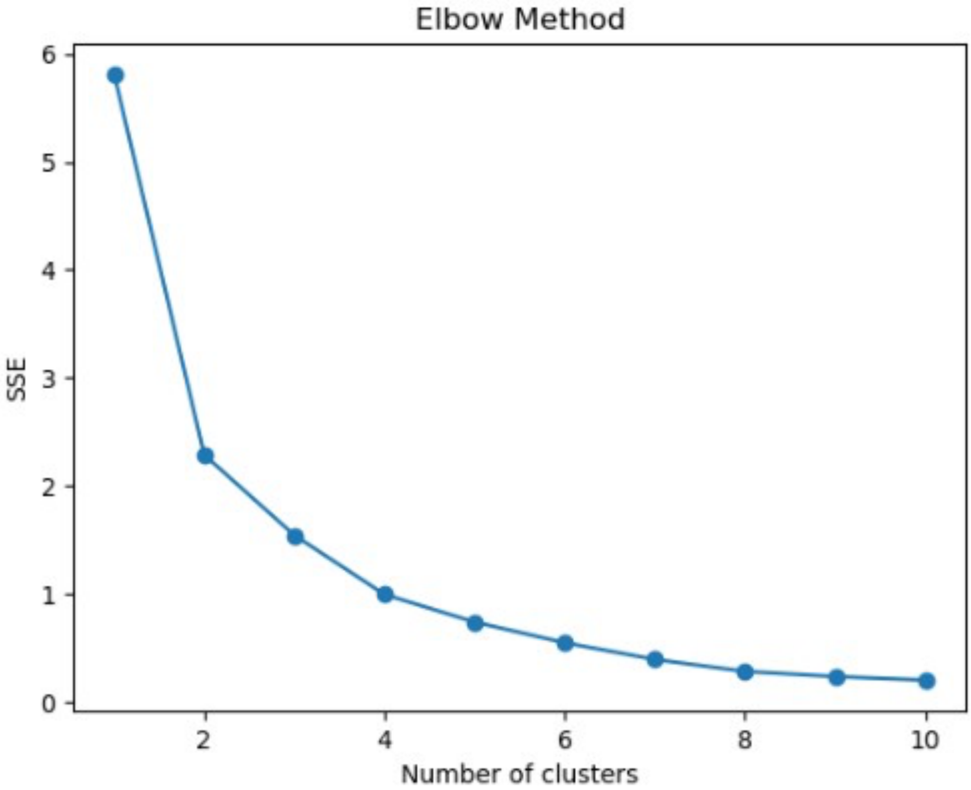

Figure S3. Determination of the optimal number of clusters using the elbow method.

The y-axis indicates the sum of squared errors (SSE) within clusters, and the x-axis indicates the number of clusters. The elbow point at k = 4 suggests an optimal clustering solution.
